# Male commuters in north and south England: risk factors for the presence of faecal bacteria on hands

**DOI:** 10.1186/1471-2458-11-31

**Published:** 2011-01-12

**Authors:** Laura Dodrill, Wolf-Peter Schmidt, Emma Cobb, Peter Donachie, Valerie Curtis, Mícheál de Barra

**Affiliations:** 1 Department of Disease Control, London School of Hygiene and Tropical Medicine, London, UK

## Abstract

**Background:**

A previous study found that the prevalence of contamination with bacteria of faecal-origin on the hands of men differed across UK cities, with a general trend of increased contamination in northern cities. The aim of this study was to (1) confirm the north-south trend (2) identify causes for the trend.

**Methods:**

Hand swabs from commuters (n = 308) at train stations in 4 cities were tested for the presence of faecal bacteria.

**Results:**

The prevalence of hand contamination with faecal bacteria was again higher in cities in the north compared to the south (5% in London, 4% in Birmingham, 10% in Liverpool and 19% in Newcastle). Contamination risk decreased with age and better personal hygiene (self-reported). Soil contact and shaking hands increased contamination with faecal bacteria. However, in multivariable analysis, none of these factors fully explained the variation in contamination across cities.

**Conclusion:**

The study confirmed the north-south differences in faecal contamination of hands without finding a clear cause for the trend. Faecal contamination of hands was associated with personal hygiene indicators suggesting that microbiological testing may contribute to evaluating hygiene promotion campaigns.

## Background

Total morbidity (both reported and the estimated unreported cases) for intestinal infectious disease within England has been estimated at 9.4 million cases per year, which scales to one person in five per year [1]. The Financial Services Authority estimated the cost of this burden to the UK economy at £350 million per year [2]. Faecal-oral carriage via the hands is thought to be an important transmission pathway for diarrhoea pathogens. The promotion of improved hand hygiene continues to be a key public health goal both in low and high income countries, in communities and in health care settings. Most previous research has been done into specific populations such as primary carers and healthcare workers [3-6], but little work has been done to investigate the carriage rate of the general population [7-9].

Normal bacterial flora of the gastrointestinal tract includes a vast array of anaerobes (organisms that exist in the absence of oxygen), gram negative organisms of the family *Enterobacteriaceae *as well as gram positive *Enterococci*. Commonly found species of *Enterobacteriaceae *are *Escherichia coli *(*E. coli*), *Klebsiella, Enterobacter *and *Proteus*. These and *Enterococcus *are known as normal flora but on occasion can cause wound and urinary tract infections. *Enterobacteriaceae *are characterised by their ability to grow on bile containing agar and their inability to produce the enzyme 'oxidase'. *Enterococci *are characterised by black colonies on bile aesculin agar and the presence of streptococcal D antigen. The importance of finding any of the above organisms on a swab sample from hands is that they are is a potential indicator of faecal contamination, which may allow the transfer of more pathogenic organisms such as *Salmonella*.

Studying the contamination of hands with pathogens may help to identify populations at risk for person-to-person spread of diarrhoea and to monitor the success of hand hygiene promotion campaigns [10]. We previously reported the prevalence of contamination with faecal bacteria on hands in the general population in England and found a conspicuous north-south trend in males, suggesting an increasing risk of contamination the further north samples were taken [11]. Here we present the results of a follow-up study with the aims of (1) confirming the north-south trend observed in the first study, and (2) exploring potential causes for the trend including differences in hygiene practices, contact patterns, animal and soil contact as well as age.

## Methods

To replicate the conditions of the original study, samples were taken at the same locations: London Euston, Birmingham New Street, Liverpool Lime Street, and Newcastle Central stations. The samples were collected by first author, LD, in all four locations. Any man appearing over the age of 16 and not engaged in any other activity (eating, telephone call, etc) was approached and asked if he wished to participate in the study. For those who agreed, sterile cotton swabs moistened with sterile water were run once up and down the pad side of each finger and thumb of their dominant hand. The swab was replaced into the charcoal transport tube (Transwab^® ^MW171 transport medium charcoal; Medical Wire & Equipment, England), and numbered. The participant was then asked to fill out the questionnaire. Samples were taken on Mondays in July 2009 between 9 am and 5 pm with the exception of Liverpool when samples were taken on Wednesday between 9 am and 5 pm. Ethical approval for the study was granted by the London School of Hygiene and Tropical Medicine Ethics committee.

### Culture of organisms

All equipment was purchased sterile or autoclaved at 121°C for 15 minutes. Media were made up according to the manufacturer's instructions. Samples were placed in glass universals containing purple MacConkey broth and an inverted glass Durham tube for gas collection. Universals were incubated for 48 hours at 37°C and those that emitted gas and turned from purple to yellow were identified as *E coli*.

2.5 ul of this broth was then streaked onto plates of (a) MacConkey #3 agar and (b) Bile Aesculin agar using a sterile loop. These plates were then incubated at 37°C for 48 hours. Where mixed colonies were observed, the growth was re-streaked, and incubated under the same conditions in order to isolate single colonies. Oxidase positive plates on the MacConkey agar plates were identified as of non-faecal origin. Oxidase negatives were tested on an API 20E. Any black colonies on Bile Aesculin were tested for the activity of catalase. Catalase positive organisms with no growth on MacConkey #3 agar were ignored as being of non-faecal origin. Catalase negative or mixed colonies were tested with Streptococcal group D antisera. The media and antisera used in the study were sourced from Oxoid (UK).

### Questionnaire

Participants completed a questionnaire with questions on age, gender, physical contact with soil, children or pets, and hand hygiene, see Table 1. The hygiene questions were extracted from the hygiene inventory[12] and were scored on a 1-4 scale where higher scores indicate better hand hygiene. These items were summed to create a composite hygiene score. In previous research, the hygiene inventory has demonstrated good reliability (internal and test-retest) and validity (positive correlation with handwashing behaviour, negative correlation with rates of infection)[12].

**Table 1:** Hygiene and risk behaviour questions

**A. Hygiene inventory: handwashing items**
1. On an average day, approximately how many times do you wash your hands?
2. Upon getting home, do you wash your hands straight away?
3. After washing your hands, do you dry your hands completely?
4. When soap is available, do you wash your hands with soap?
*5. Do you use antibacterial gel or wipes to clean your hands?*
**B. Risk behaviour questions**
6. Have you touched a pet today?
7. Have you touched any soil or gardening apparatus/tools today?
8. Have you had any contact with any children less than five years old this morning?
9. Have you shaken hands with anyone today? If yes, approximately how many people?

Note: Item 1 had the following response options: "never" (scored as 0), "1 to 5" (1), "6 to 10" (2), and "11+" (3). Items 2 - 5 had the following response options: "always" (3), "usually" (2), "occasionally" (1), and "rarely" (0). Items 6 - 8 had a yes/no response format. Item 9 also had a box to enter number of handshakes.

### Statistical analysis

Categorical and binary variables were compared between the four study sites using the chi-square test. Continuous variables were compared using an ANOVA. The hygiene score was compared across sites using the Kruskal Wallis test. Finally we performed a logistic regression analysis to determine which variables affect the likelihood of contamination being present on the hands. For this procedure we retained variables based on backward elimination, p < 0.1.

## Results

In total, 308 males agreed to participate in the study, with a mean age of 36.2 years. The characteristics of study participants in different sites are listed in Table 2. As use of the underground train was specific to London, method of travel was divided into bus, train (including over- and underground), other, and none (i.e., walking/cycling). There were no great differences in the hygiene scores across sites (Table 2). We explored the association between different factors and the hygiene score (as below vs. above the median hygiene score) using logistic regression. Neither age, location, contact with soil, pets, nor living with children were found to be associated with the score.

**Table 2:** Characteristics of the study population

		London (n = 80)	Birmingham (n = 76)	Liverpool (n = 69)	Newcastle (n = 83)	P-value
		n (%)	n (%)	n (%)	n (%)	
Method of transport						<.001
	None	0 (0)	18 (23.7)	16 (23.2)	12 (14.5)	
	Bus	10 (12.5)	45 (59.2)	24 (34.8)	34 (41)	
	Train	65 (81.25)	13 (17.1)	29 (42)	32 (39)	
	Other	4 (5)	0 (0)	0 (0)	5 (6)	
Mean age:		35.8	35.4	34.7	38.6	.26
Occupation						.30
	Professional	37 (46.3)	29 (38.2)	17 (24.6)	28 (33.7)	
	Manual	26 (32.5)	32 (42.1)	38 (55)	35 (42)	
	Student	11 (13.75)	11 (14.5)	9 (13)	12 (14.5)	
	None	6 (7.5)	4 (5.3)	5 (7.3)	8 (9.6))	
Median hygiene score		8	7	7	7	.22.
Soil contact		2 (2.5)	10 (13.2)	4 (5.8)	12 (14.5)	.02
Living with children		10 (12.5)	10 (13.2)	8 (11.6)	19 (22.9)	.16
Average number of handshakes		1.4	0.7	1.3	1.4	.1
Pet ownership		14 (17.5)	15 (19.7)	12 (17.4)	10 (12.1)	.9

Overall, 10% (N = 31) of the swabs taken tested positive for faecal coliforms. Table 3 shows the identified bacteria by site. Overall, 5% of samples tested positive for *E. coli *and 4% for *enterococcus *sp. *Pantoea *and *Klebsellia *were much less common. Isolation of *E. coli *was particularly common in Newcastle. As in our previous study, we found an increasing contamination in northern cities (Liverpool and Newcastle) compared to the southern sites (London and Birmingham). In fact, the differences between the study sites appeared to be explained by differences in *E. coli *isolation. No samples tested positive for multiple faecal coliforms.

**Table 3:** Bacteriological test outcomes

Faecal Bacteria		Overall	London	Birmingham	Liverpool	Newcastle
		n (%)	n (%)	n (%)	n (%)	n (%)
Any faecal bacteria		31 (10.1)	4 (5)	4(5.3)	7 (10.1)	16 (19.3)
	*Enterococcus*	11 (3.6)	3 (3.8)	1 (1.3)	3 (4.3)	4 (4.8)
	*Enterobacter amnigenus*	1 (0.3)	0 (0)	1 (1.3)	0 (0)	0 (0)
	*Enterobacter cloacae*	1 (0.3)	0 (0)	0 (0)	0 (0)	1 (1.2)
	*Klebsiella*	1 (0.3)	0 (0)	0 (0)	1 (1.4)	0 (0)
	*E. coli*	14 (4.5)	0 (0)	0 (0)	3 (4.3)	11 (13.3)
	*Pantoea*	3 (1.0)	1 (1.3)	1 (1.3)	1 (1.4)	0 (0)
No faecal bacteria		277 (89.9)	76 (95)	73 (94.7)	62 (89.9)	66 (80.7)

The univariate analysis of factors associated with faecal hand contamination is shown in Table 4. Relative to professional workers, students were more likely to show contamination on hands. Odds of contamination declined with age and increased with the number of handshakes that day. Favourable hygiene scores were associated with lower contamination risks. Analysis of the individual items of the hygiene score did not suggest that some items were more associated with hand contamination than others. In general we found a negative association between scores of each item of the hygiene score and hand contamination (not shown). Soil contact was associated with higher contamination risk, while living with children and pet ownership were not.

**Table 4:** Univariate logistic regression analysis of factors associated with faecal contamination

Factor		Number contaminated (% contaminated)	OR	95% CI
Location				
	London	4 (5)	1 (ref)	-
	Birmingham	4 (5.3)	1.1	0.3 - 4.4
	Liverpool	7 (10.1)	2.1	0.6 - 7.7
	Newcastle	16 (19.3)	4.5	1.4 - 14.2
Occupation				
	professional	7 (6.3)	1(ref)	-
	manual	12 (9.2)	1.5	0.6 - 3.9
	student	12 (27.9)	5.8	2.1 - 15.9
	none	0 (0)	-	-
Age				
	16-25	17 (21.3)	1 (ref)	-
	26-35	9 (10.3)	0.4	0.2 - 1.0
	36-45	2 (3.3)	0.1	0 - 0.5
	46 +	3 (4)	0.2	0 - 0.6
Hygiene score				
	0-4	11(21.6)	1 (ref)	-
	5-7	14 (12.5)	0.5	0.2 - 1.2
	8-10	5 (4.8)	0.2	0.1 - 0.6
	11+	1 (2.5)	0.1	0.0 - 0.8
Number of hand shakes on day of recruitment				
	0	16 (9.6)	1 (ref)	-
	1-3	7 (6.4)	0.6	0.3 - 1.6
	4-6	3 (15.8)	1.9	0.6 - 6.2
	7 +	4 (50)	9.4	2.2 - 41.4
Soil contact				
	No	24 (8.5)	1 (ref)	-
	Yes	7 (25)	3.6	1.4 - 9.2
Contact with children				
	No	27(10)	1 (ref)	
	Yes	4 (9)	0.8	0.3 - 2.4
Pet ownership				
	No	2 (10)	1 (ref)	-
	Yes	4 (10.5)	1.1	0.4 - 2.7

*Note*: OR: Odds Ratio, CI: Confidence interval.

The multivariate analysis largely confirmed the results of the univariate analysis, see Table 5. In cities further to the north the odds of contamination was higher than in southern cities (London and Birmingham), but the odds ratios were slightly lower compared to the univariate analysis. Thus, the variables included in the model only marginally explained the north-south differences between study sites.

**Table 5:** Multivariate logistic regression analysis of factors associated with faecal contamination

Factor		OR	95% CI
Location			
	London	1 (ref)	-
	Birmingham	0.7	0.2 - 3.5
	Liverpool	1.5	0.4 - 5.8
	Newcastle	3.5	1.0 - 12.1
Age *		0.9	0.9 - 1.0
Hygiene score			
	0-4	(ref)	-
	5-7	0.6	0.2 - 1.7
	8-10	0.2	0.1 - 0.7
	11+	0.1	0.05 - 0.7
Number of hand shakes on day of recruitment*		1.2	1.0 - 1.5
Soil contact		5.4	1.7 - 16.9

*Note*: OR: Odds Ratio, CI: Confidence interval. *OR denotes relative increase in odds with every additional unit increase (i.e. year, number of handshakes)

## Discussion and Conclusions

Our data replicate the finding [11] that male hand contamination rates differ between UK cities, with higher rates of contamination in northern than the southern cities studied. Men sampled in Liverpool were twice as likely to have bacteria of faecal-origin on their hands as males in Birmingham or London. In Newcastle, men were more than four times more likely to test positive than men from southern cities. It is unclear whether higher contamination risk also translates into high risk of infectious intestinal disease. Surveillance data comparing northern and southern regions in England tend to find higher rates in the south [13].

A number of variables besides location predicted hand contamination rates. Men who shook hands more often had a greater likelihood of contamination, presumably due to spreading contamination [7]. Similarly, men who had engaged in soil contact (for example, through sporting activities or gardening) were more likely to be contaminated, most likely because of prior contamination of soil with animal or human wastes, or faecal bacteria that naturally also occur in the soil.

Lower hygiene scores - as measured by the items from the hygiene inventory [12] - were associated with greater likelihood of contamination. While previous research has demonstrated correlations between scores and actual hygiene behaviour in an experimental context [12], these data provide evidence that hand hygiene practices as measured by this scale are associated with levels of hand contamination, even though self-reported hygiene practices have been found unreliable as absolute figures [14,15]. Psychometrically validated scales like that generated by Stevenson *et al. *are thus a promising tool for future research on hand hygiene behaviour.

The association between hand contamination and age, independent of other factors controlled for in the multivariate analysis, was unexpected. This effect may be due to increasing hygiene across the lifespan, or, alternatively, differences in lifestyle and behaviour not captured by the questionnaire.

These factors - age, hygiene, hand-contact, or soil contact - explain only a small part of the between-city trend in hand contamination rates. Possibly, we did not measure the existing variables with sufficient accuracy to exclude residual confounding. More likely, however, there could be other differences in hygiene, lifestyle and behaviour explaining the trend that our questionnaire did not cover. Possible explanations include differences in climate, type of occupation or leisure-time activities. It is possible that differences in normal skin flora rather than hygiene may resulted in longer survival rates of faecal contaminants, and thus higher contamination rates in northern cities. Whether the unexpected more frequent isolation of *E. coli *in Newcastle compared to other cities was due to chance or represents a true difference between the study sites could be the subject of future studies. In our previous study the differences in contamination between the sites were due to *enterococcus *as well as *E. coli*.

While bacteria like *E. coli *and *Enterococcus faecalis *are known to cause disease, the presence of some of the other identified bacteria on hands like *Pantoea *may not necessarily be a health hazard in itself. In any case, the presence of these bacteria appear to be indicative of a failure of good hygiene practice, and more specifically, of a failure to wash hands after contact with faecal material. Alternatively, these bacteria may have been picked up by touching a surface which someone with poor post-defecation hygiene had previously touched. The faecal indicator bacteria identified in this study may therefore be useful to study trends in the overall hygiene behaviour of a population. It is interesting to note that the overall contamination prevalence in this survey was markedly lower than in the previous study in all cities [11]. The present survey was conducted in June and July 2009, coinciding with the first wave of the H1N1 influenza pandemic, and amid large scale public health campaigns emphasizing the role of hand hygiene for influenza control (the previous survey was done in August and September 2008). Increased handwashing and use of hand sanitisers may have contributed to the lower prevalence of faecal bacteria on hands, although other factors, such as temperature, cannot be excluded. Larger studies over a longer time scale are needed to confirm whether hand contamination is a useful surveillance tool for hand hygiene behaviour.

The study was conducted in the same sites as the previous study [11] in order to replicate the findings. Future studies could include different cities from the north and south of England to exclude the possibility that the observed trends may be due to specific characteristics of the included cities, independent of the geographic location.

This study did not attempt to quantify the number of bacteria that were found on hands; hence hands could be reported as positive with only one or very few bacteria. We also did not check for the presence of *Pediococci *or *Aerococcus viridians*, which may have been a source of false-positives for *Enterococci*. It should be noted that although the bacteria we isolated are likely to be of faecal origin, *Enterobacter, Pantoea*, and *Klebsiella *may have been picked up from non-faecal sources, e.g. from working with food or animals. However, as these were isolated in only a small number of our samples, the general pattern of our results are unlikely to be affected.

In conclusion, this study confirmed the higher prevalence of hand contamination in northern cities but failed to identify a reason for this trend. The study also confirmed that the contamination of hands with faecal bacteria is associated with typical risk behaviours for person-to-person transmission of pathogens (hand hygiene, shaking hands, soil contact, etc.). As unbiased estimates of risk behaviour, especially hand hygiene, can be difficult to obtain, microbiological methods could contribute to the study of hygiene behaviour [10].

## Competing interests

MdB, VC and WPS receive funding from Unilever. However Unilever played no role in the design, implementation or interpretation of this study, and their association with these authors is described only in order to maintain transparency.

## Authors' contributions

LD collected the data. VC conceived of the study and LD, MdB and VC designed the study. LD, EC, and PD analysed the samples. LD, MdB and WPS analysed the data. WPS and MdB composed the manuscript. All authors read and approved the final manuscript and authors' contributions section.

## Pre-publication history

The pre-publication history for this paper can be accessed here:

http://www.biomedcentral.com/1471-2458/11/31/prepub
